# Pulmonary function in swimmers exposed to disinfection by-products: a narrative review

**DOI:** 10.3389/fphys.2024.1473302

**Published:** 2025-01-06

**Authors:** Michał Boraczyński, Tomasz Balcerek, Nikola Rożkiewicz, Monika Pabiszczak, Michał Harasymczuk, Aneta Sławska, Przemysław Lutomski

**Affiliations:** ^1^ Department of Physiotherapy, Faculty of Health Sciences, Collegium Medicum, University of Warmia and Mazury in Olsztyn, Olsztyn, Poland; ^2^ Department of Gynecology Obstetrics and Gynecologic Oncology, Gynecological Obstetric Clinical Hospital of Poznan University of Medical Sciences, Poznań, Poland; ^3^ Medica Pro Familia, Family Medicine Clinic, Non-public Healthcare Center, Poznań, Poland; ^4^ MALTA Family Medicine Clinic, Non-public Healthcare Center, Poznań, Poland; ^5^ Department of Traumatology, Orthopedics and Hand Surgery, Poznan University of Medical Sciences, Poznań, Poland; ^6^ Department of Sport Medicine and Traumatology, Poznan Univeristy of Physical Education, Poznań, Poland

**Keywords:** respiratory system, asthma, swimming, chlorine-based disinfectants, β2-agonists

## Abstract

Swimming produces many psychophysiological effects, including blood, hormonal, enzymatic, pulmonary, cardiovascular and energetic adaptations. However, asthma and allergies are becoming increasingly prevalent medical issues among elite endurance-trained swimmers, where exercise-induced asthma or bronchospasm is frequently reported. Heavy endurance swimming training, especially under adverse conditions, stresses the airway mucosa, leading to inflammatory changes, as observed in induced sputum in competitive swimmers. In addition, chlorine-based disinfectants (CBDs) are commonly used in indoor pools due to their effectiveness and lower relative cost. Many of these substances have carcinogenic and genotoxic properties, and exposure to DBPs have been linked to adverse respiratory effects. The association between long-term exposure to a chlorinated swimming pool and elevated serum sIgE levels suggests a link between allergens, chlorine exposure and the development of various pulmonary dysfunctions. Thus, the combination of intense and repeated physical endurance training over extended periods, along with suboptimal environmental conditions, may contribute to the development of rhinitis, asthma and bronchial hyperresponsiveness in athletes. While occasional or low-level exposure to chlorine might not be harmful, regular swimmers, especially those at competitive levels, are at a higher risk of developing respiratory disorders. Because these potential risks of exposure to CBDs must be balanced against the benefits of swimming and the risk of microbial infections in pools, we recommend better pool management and regular health checks for swimmers. Fortunately, the reduction of bronchial symptoms in swimmers who reduce training volume and intensity suggests that the negative effects on pulmonary function may be reversible. For these reasons, it is crucial to develop effective respiratory protection strategies, including medical interventions and modifications to the pool environment. Practical steps such as reducing chlorine use, ensuring proper hygiene before swimming and using swim caps can minimise risks. Research should also explore safer alternatives to CBDs, such as ozonation, and improved ventilation to reduce air pollutants.

## Introduction

Swimming is recognized as one of the most effective training methods for enhancing physical activity and fascilitating healthy aging across the general population (Costello et al., 2019). With its multitude of benefits, it is highly favored as a versatile and highly physiological type of muscle activity and sport for people of all ages. It is often ordered for patients with mobility impairment, allergic and respiratory diseases (Font-Ribera et al., 2014; Cavaleiro et al., 2018). Intense swimming workouts are recommended to increase cardiorespiratory capacity in athletes (Gonjo and Olstad, 2021). It also has a positive influence on lung, blood and heart volumes (also optimizes vascular tone), immune system as well as maximal oxygen uptake (V̇O_2_max) and aerobic capacity (Morgado et al., 2020; Hadiansyah et al., 2022; Wu, 2022). For this reason the lung physiology of swimmers has been a subject of long-standing interest, with the earliest studies dating back to the 1960s (Astrand et al., 1963; Andrew et al., 1972; Courteix et al., 1997; Bovard et al., 2018; Rochat et al., 2022).

However, competitive and endurance swimming can affect respiratory health, causing an increase in airway reactivity and the incidence of exercise-induced asthma (EIA), exercise-induced bronchospasm (EIB) and other respiratory symptoms over the years (Mountjoy et al., 2015; Cavaleiro et al., 2018).

These pro-asthmatic changes have been associated with airway hyperresponsiveness (AHR), airway epithelial remodelling and damage as shown in bronchial biopsies studies (Bougault et al., 2012). It may be caused by the regular exposure to chlorine-based disinfection by-products (Bougault et al., 2009; Cavaleiro et al., 2018; Mountjoy et al., 2015; Schittny, 2017; Hsia, 2004). These products might be inhaled, ingested or absorbed via skin during the swimming practice (Cavaleiro et al., 2018; Belda et al., 2008; Jacobs et al., 2007).

The debate over the reasons for the observed differences between swimmers and other athletes continues, mainly due to the expectation that structural changes in swimmers’ airways will result in reduced expiratory flow rates. Contrary to this expectation, swimmers often demonstrate superior lung function parameters (Armour et al., 1993; Sable et al., 2012; Boulet and O'Byrne, 2015; Lazovic-Popovic et al., 2016; Schittny, 2017). According to a study by Lazovic-Popovic et al. (2016), swimmers had statistically significantly higher VC, FVC and FEV1 (measured in L) and FEV1/FVC ratio compared to football players (all *p* values <0.001).

This phenomenon may be enhanced by the regular training that most swimmers undertake for many years during the growth period, when the lungs are also expanding very intensively. New research on pulmonary development shows that continuous allveolarization and microvascular maturation of the capillary bed takes place until early adulthood (Schittny, 2017; Hsia, 2004). Therefore, there are specific implications of exercise and training in an aquatic environment, as adaptive mechanisms to intensive swimming training may affect the evolution of airways and alveolar spaces, leading to enhanced lung growth (Rochat et al., 2022).

### Respiratory disorders in swimmers

Asthma, derived from the Greek word ἆσθμα (âsthma) which means “panting” (Murray, 2010), is a variable, long-term condition that affects over 350 million children, adolescents and adults worldwide (Meghji et al., 2021), and 250,000 deaths per year are attributed to the disease. It is expected that this amount will reach more than 400 million by 2025 (Masoli et al., 2004). From a medical standpoint, asthma is a complex, heterogenous and chronic inflammatory disease that affects the airways of the lower respiratory system. The constriction of the airways varies between individuals and is caused by excessive T-helper type 2 (Th-2) cell responses and specific immunoglobulin E (IgE) antibodies production, which trigger inflammation-related increases in bronchial muscle tone (Kuruvilla et al., 2019; Ray et al., 2021). The mechanisms causing asthma development remain not fully understood but are believed to involve a co-occurrence of inflammation and airway remodelling, ultimately leading to bronchial hyperresponsiveness. Sensitizer-induced asthma often presents with elevated eosinophil levels in blood and sputum, alongside activation of T-helper type 2 (Th2) cells. This activation triggers the release of inflammatory cytokines (i.a., IL-4, IL-5, and IL-13) and promotes IgE synthesis. Occupational exposures are a common cause of sensitizer-induced asthma (Clausen et al., 2020; Tarlo and Lemiere, 2014). A plethora of factors cause the disease, provoke asthma exacerbations, and contribute to asthma disease progression (Kuruvilla et al., 2019). The incidence of asthma depends on factors including age, geographical location, socioeconomic status, and race. Moreover, the factors that contribute to the progression to asthma from viral- and allergen-induced respiratory symptoms are largely unexplored. The primary symptoms of asthma are coughing, wheezing, and dyspnea, with additional signs encompassing bronchospasm, chest tightness, mucous edema, and sputum production. Asthma symptoms can vary in intensity and severity, which can affect daily activities (Clearie et al., 2010).

Acute bronchial and laryngeal symptoms development is influenced by hereditary and external factors, such as air pollution, tobacco smoke, viral infections, and exposure to allergens. Certain medications, such as angiotensin-converting enzyme inhibitors, aspirin, and non-steroidal anti-inflammatory drugs (NSAIDs), may also cause asthma (Covar et al., 2005). Other factors that can trigger asthma symptoms include common substances of animal and vegetable origin, as well as substances that cause immune responses. However, the most significant factors that produce asthma symptoms are house dust mites, animal dander (fragments of fur or feathers), cockroaches, fungi, mould, yeast, and pollen allergens (Voisin et al., 2010; Joyner et al., 2006). The development of asthma is influenced by both genetic predispositions and environmental factors. Genetic factors involve immune responses to small doses of allergens, which can cause oversecretion of IgE antibodies against these allergens (allergic sensitization). In addition, blood eosinophilia (elevated blood eosinophil counts) correlates with airway eosinophilic inflammation in patients with asthma (Pignatti et al., 2018). The latter pathological condition is stimulated by the adaptive and/or innate immune system: Th-2 cells and type-2 innate lymphoid cells (ILC2s), respectively. Thus, multiple asthma-associated single nucleotide polymorphisms (SNPs) may constitute genetic susceptibility to eosinophilic asthma (El-Husseini et al., 2023).

In recent years many scientists have shown the influence of chlorine disinfectants and risks of new-onset allergic sensitization and airway inflammation after early age swimming in chlorinated pools. To be triggered, these interactions between pool chlorine and atopy require prior sensitization to aeroallergens, as well as cumulative exposure to chlorinated pools over a period of at least a few years (Voisin et al., 2014). Although chemical exposures from disinfectant products used on swimming pools often go unnoticed or unrecognized, they have been linked to asthma-related symptoms and exacerbations. Individuals with atopy are hyperallergic to substances that are otherwise harmless to the healthy population. While some people with asthma rarely experience symptoms, others may react frequently and readily and experience persistent symptoms. Clinical research indicates that the risk of asthma is 48%–79% higher in genetically predisposed individuals (El-Husseini et al., 2023). Unfortunately, both maternal and paternal disease state affects offspring disease, and based on individual studies, maternal asthma is the more potent contributor (Guilbert et al., 2007). For example, children of asthmatic or atopic parents have a 3 to 6 times greater risk of developing asthma (Lim et al., 2010). In recent years, there has also been observed a correlation between obesity and an increased risk of asthma (Beuther, 2010; Holguin and Fitzpatrick, 2010). For example, Sutherland et al. (2008) found in 30 asthmatic women before and after methacholine provocation that the increase in functional residual capacity and decrease in inspiratory capacity were significantly greater in obese asthmatic women (*p* < 0.001 and *p* = 0.003, respectively). Additionally, some genetic variants may only cause asthma when combined with specific environmental exposures (Martinez, 2007). Genetic predispositions may contribute to the improper function of beta-2-adrenergic receptors (ADRB2, also known as β2-AR), which can reduce their activity and cause disturbed tonicity of bronchial muscles due to the dominance of the parasympathetic nervous system controlling muscle contraction (Liang et al., 2014).

## Pathophysiological background of EIA and EIB

Exercise-induced asthma is a type of inflammatory condition in which individuals with diagnosed bronchial asthma experience constriction of the lower airways and other asthma symptoms induced by physical exercise. This specific medical condition is a common concern, especially for growing children, with studies showing that it occurs in 70%–90% of asthma patients (Courteix et al., 1997). On the other hand, EIB is defined as the transient and reversible narrowing of bronchi that occurs during or after exercise, and it is observed in both healthy and symptomatic subjects (i.e., asthmatic patients). In subsequent years, EIB was separated into EIB with asthma and EIB without asthma (Parsons et al., 2013), the latter being particularly common in athletes (Carlsen et al., 2008). The main symptoms include wheezing, dyspnea, cough, and chest tightness (Aggarwal et al., 2018). The EIA and EIB are often used interchangeably to describe symptoms of asthma, but the main difference between the terms is that an asthma attack can occur during other activities, while EIB occurs only during or after submaximal and/or vigorous exercise (Aggarwal et al., 2018; Goossens et al., 2022). Unfortunately, only at least of 5–8 min continuous high-intensity exercise is required to develop an EIB response in children with asthma (van Leeuwen et al., 2011). After exercise, EIB resolves spontaneously within 1 h (Smoliga et al., 2016).

Pathogenic mechanisms of EIA/EIB probably differ in the athlete compared to children, adolescent, or adult with asthma (Carlsen, 2012). Until now, the pathomechanism of EIB has not been fully explained, but it is thought to involve osmotic (or airway drying), vascular (or thermal), and epithelial microtrauma mechanisms (Pigakis et al., 2022).

The incidence of EIB varies among adult athletes, with different studies reporting rates ranging from 30% to 70% (Goossens et al., 2022). Damage of airway epithelium, overexpression of cysteinyl leukotrienes, reduced protection from prostaglandin E2, and heightened airway eosinophilia have been identified as distinct immunopathologic features of asthma with exercise-induced bronchoconstriction, validating its inflammatory basis. Studies have demonstrated a link between the presence of columnar epithelial cells in induced sputum and the severity of EIB, as well as a correlation between the concentration of these cells and the levels of histamine and cysteinyl leukotrienes in the airways, confirming the role of mediator release. Vigorous exercise leads to the inhalation of large volumes of cold, dry air, triggering key pathogenic mechanisms of EIB, including airway osmotic changes, epithelial damage, inflammation, and neuronal activation (Couto et al., 2018).

The vascular hypothesis proposes that increased pulmonary ventilation during exercise causes the loss of heat in the airways. This cooling triggers a reflexive expansion of blood vessels and their hyperaemia, aimed at warming the linings of the bronchial tree, followed by a constriction of the airways (Parsons et al., 2008; Thickett et al., 2002). The third theory is the theory of epithelium micro-trauma. The mechanism here involves small airway epithelium dehydration combined with exposure to shear stress caused by increased airflow and increased transepithelial pressure gradient. This may cause disruption and injury to the epithelial cells, leading to airway remodelling. Another factor in epithelium microtrauma is air quality. Training indoors *versus* outdoors affects exposure to allergens and air pollution (Parsons et al., 2008; Thickett et al., 2002).

### Prevalence of the respiratory tract symptoms

In recent years, concerns have been raised regarding the potential negative effects of water disinfectants used in swimming pools on the respiratory system and the development of hypersensitive reactions among regular swimmers (Manasfi et al., 2017; Yang et al., 2018).

A high frequency of upper and lower airway respiratory symptoms has been reported in competitive athletes and school children attending regularly to the swimming pools (Hsia, 2004; Belda et al., 2008; Momas et al., 1993). These results are summarised in Table 1. Several studies have shown that nasal symptoms were highly frequently in swimmers regularly attending pools, mainly 7%–74% of swimmers complaining of chronic rhinitis symptoms (Murray, 2010; Deitmer and Scheffler, 1990; Gelardi et al., 2012). Moreover, even 44% of young competitive swimmers with rhinitis had a pre-existing allergic component (Gelardi et al., 2012). Given the relatively high prevalence of viral infections in competitive swimmers, nasal symptoms may be caused by an acute viral infection or a post-infection condition (Gleeson et al., 2002; Moreira et al., 2009; Gleeson and Pyne, 2016). From a general perspective, the major upper respiratory symptoms reported are itching, sneezing, nasal obstruction, rhinorrhoea and symptoms associated with sinusitis (Deitmer and Scheffler, 1990; Potts, 1994; Gelardi et al., 2012).

**TABLE 1:** Prevalence of respiratory tract symptoms.

Citation	n	Cough	Wheezing	Breathless-ness	Chest tightness	Chest congestion	Sneezing/Itching	Rhinorrhea	Nasal obstruction	Sinusitis	Throat irritation	Eye irritation	Headache
Font-Ribera et al. (2014)	2758		1064					764					
Zwick et al. (1990)	14							6			1	4	
Momas et al. (1993)	210	50					92	130			23	34	40
Deitmer and Scheffler (1990)	20						8	8	6	8			
Gelardi et al. (2012)	44							33					
Potts (1994)	738	266	192	288	185	170	332				199	266	266
Levesque et al. (2006)	305	79	27	73		27	91				58	113	88
Helenius et al. (2002)	16		10–13					10					
Langdeau et al. (2000)	25	6		4		14							
Turcotte et al. (2003)	95	10	10	31	8								11
Päivinen et al. (2010)	332	90	71	142									
Zaccarin et al. (2022)	396	167	67	99	75		247	186			99		
Seys et al. (2015a)	41	19		20				3	3			11	
Bernard et al. (2006)	341	62	32	24	19								
Font-Ribera et al. (2009)	3223		319					1116				1111	
Paciência et al. (2021)	33							10					

Swimmers also report ocular symptoms, throat pain and headache (Potts, 1994; Levesque et al., 2006). Both upper and lower airway respiratory symptoms may have a noxious influence on swimmers performance. Zwick et al. (1990) reported that 79% of the swimmers training 27–37 h per week presented various upper and lower respiratory symptoms compared with 21% of controls. Helenius et al. (2002) observed swimming-induced lower airway respiratory symptoms in 57% of the 42 Finnish National Team swimmers. Both studies of Turcotte et al. (2003) and Levesque et al. (2006) showed a clear predominance of symptoms such as cough, wheezing, breathlessness and chest congestion. Interestingly, the symptoms of exercise-induced breathlessness shown in the study by Turcotte et al. (2003) were more often reported by female (42.7%) than male athletes (29.6%, *p* = 0.0004). Seys et al. (2015a) and Bernard et al. (2006) reported in their research that the main symptoms observed were: cough (46% and 18%, respectively) and breathlessness (49% and 7%, respectively).

However, Deitmer and Scheffler (1990) observed only upper respiratory symptoms such as sneezing, itching, nasal obstruction and sinusitis while there were no symptoms as rhinorrhoea, nasal obstruction or sinusitis reported by Potts (1994). Although Potts (1994) noticed that among various symptoms we can indicate major ones, as, for example,: sneezing/itching (45%), breathlessness (39%), cough (36%), wheezing (36%), and/or headache (36%). Päivinen et al. (2010) investigated that 43% of top Finnish swimmers reported breathlessness and 27% submitted cough. On the other hand, Font-Ribera et al. (2009) showed that among 3223 participants, 35% developed rhinorrhea and about 35% had eye irritation, while another study showed that among 2758 participants, 39% developed wheezing and 28% rhinorrhea (Font-Ribera et al., 2010).

Respiratory symptoms are not only frequently reported by elite swimmers, but also by children and youths regularly attending to the swimming pools. However, Paciência et al. (2021), Langdeau et al. (2000), Gelardi et al. (2012) and Momas et al. (1993) observed mainly rhinorrhoea. Zaccarin et al. (2022) conducted a study on young Italian competitive swimmers, revealing a predominance of upper respiratory symptoms, with 47% experiencing nasal congestion/rhinorrhea and 62% reporting sneezing. Additionally, the most frequently observed lower respiratory issues included difficulty breathing (25%), chest tightness (19%), wheezing (17%), and coughing (42%) (Seys et al., 2015b). The most frequently reported symptoms of lower respiratory system were cough and asthma-like symptoms such as breathlessness, wheezing and chest tightness, whereas out of upper tract were presented mostly throat irritation, rhinorrhea and sneezing/itching. The most frequent non-respiratory tract symptoms were headache and eye irritation.

Swimmers have increased exposure to chlorine and other chemicals, and this exposure can induce airway sensitization (Pigakis et al., 2022). Chlorination by-products (CBPs) have a strong oxidising potential, and exposure may contribute to airway damage, causing barrier disruption (Musch et al., 2006; Bernard, 2007).

A summary of the studies describing short- and long-term changes in respiratory biomarkers are presented in Table 2.

**TABLE 2:** Studies describing changes in respiratory biomarkers.

Belda et al. (2008)		Sputum neutrophil counts	• it has been observed a correlation between the duration of training and sputum neutrophil counts in elite aquatic-based, but not nonaquatic-based athletes• this supports the idea that cumulative life time exposure to chlorine by-products is important for the development of airway dysfunction
Parsons et al. (2008)		Sputum IL-5, IL-8, IL-13, cysteinyl-leukotrienes, prostaglandin E2, histamine, leukotriene B4, and thromboxane B2 + neutrophils, lymphocytes, eosinophils, macrophages and epithelial cell counts in sputum	• Concentrations of inflammatory mediators (Cys-leu, PGE2, histamine, TXB2 and LTB4) are significantly elevated in EIB + athletes compared to EIB- athletes • The severity of EIB was significantly correlated with increased concentrations of select inflammatory mediators
Carbonnelle et al. (2002)	Chlorinated pool (NCl3 mean concentration: 490 μg/m3) for recreational swimmers Chlorinated pool (NCl3 mean concentration: 355 μg/m3) vs copper/silver pool for trained swimmers	Serum SP-A, SP-B and CC16	• CC16 was not increased in recreational swimmers • In trained swimmers CC16 peaked immediately after strenuous exercise, both in the copper/silver and in the chlorinated pools • SP-A and SP-B were unaffected by strenuous exercise in the copper/silver pool • SP-A and SP-B were significantly increased in a time-dependent manner in recreational and trained swimmers attending the chlorinated pool
Carbonnelle et al. (2008)	Chlorinated pool (NCl3 concentration: 160–280 μg/m3) vs copper/silver pool (NCl3 <20 μg/m3)	FeNO; serum SP-A, SP-B, CC16, KL−6	• FeNO increased in the copper/silver pool, whereas it did not change in the chlorinated pool, suggesting that chlorination might inhibit NO-induced vasodilation in exercise • Serum pneumoproteins were unchanged excepted SP-A which decreased after exercise in the chlorinated pool (*p* < 0.05)
Font-Ribera et al. (2010)	Chlorinated indoor pool	FeNO; serum SP-D and CC16; 8-isoprostane, several cytokines (RANTES, Ip10, TNF, IL-12p70, IL-10, IL-8, IFN-γ, and IL-4) and VEGF in EBC	• CC16 slightly increased after a swimming session • No significant changes in lung function, SP-D, 8-isoprostane, cytokines, or VEGF.
Fernández-Luna A et al. (2013)	Chlorinated pool vs ozone-treated pool	Serum SP-D and CC16	• No change was observed in lung function and SP-D in swimmers attending both pools • CC16 was significantly increased in subjects attending the chlorinated pool but not in those using the ozone- treated pool
Seys et al. (2015b)	Swimming pool	Selected panel of cytokines: Th1 (IFN-c), Th2 (IL-4, IL-5), Th22 (IL-22), epithelial cell-derived (IL-25, IL-33, TSLP) and innate (IL-1b, IL-6, CXCL8, TNF) Sputum cytokine mRNA Sputum MPO, HMGB-1, serum CC-16	• Intensive swimming for 90 min resulted in an increase of sputum IL-1β, IL-6 and TNF mRNA in competitive swimmers • Baseline sputum uric acid, high mobility group box-1, CXCL8 mRNA, sputum neutrophils and serum Clara cell protein-16 (CC-16) were significantly higher in competitive swimmers compared with controls
Font-Ribera et al. (2019)	Chlorinated pool (NCl3 mean concentration: 473 μg/m3)	Serum CC16; exhaled breath THMs; urinary TCAA; genotoxicity biomarkers (Urine mutagenicity, micronuclei in peripheral blood lymphocytes (MN-PBL), and micronuclei in reticulocytes (MN-Ret))	• Creatinine-adjusted urinary TCAA increased by 3.1 μmol/mol • Urine mutagenicity, MN-PBL, MN-Ret and serum CC16 levels remained unchanged after swimming • No correlation between CBP exposure and MN-PBL, urine mutagenicity and CC16 • Moderate associations observed for MN-Ret and CBP exposure
Kotsiou (2019)	Kotsiou (2019)	FeNO	• Postswimming FeNO values remained significantly higher in swimmers with asthma compared with those without asthma
Jonckheere et al. (2019)		CC16, serum uric acid, sputum IL-1b mRNA levels; sputum IL-8 mRNA levels	• Serum uric acid was shown to be higher in EIB + athletes compared to EIB- athletes • It was demonstrated that young athletes with sputum IL-1b mRNA levels higher than 300 are more prone to have or develop EIB • Athletes with sputum IL-8 mRNA levels lower than 190 are at very low risk of developing EIB
Bernard et al. (2008)	Outdoor chlorinated pools	IgE serum level	• Odds for asthma were significantly increased among adolescents with total serum IgE >25 kIU x L (-1), on average by 1–2 units for each 100-h increase in pool attendance • Use of residential outdoor pools was also associated with higher risks of elevated exhaled nitric oxide and sensitisation to cat or house dust mite allergens
Romberg et al. (2011)		urinary CC16	• In swimmers with a weekly training duration of 10–30 h the urinary CC16 was greater following a 6–8 min swim challenge in 90% of those sampled

### Chlorine-based disinfectants and their effect on respiratory tract

**FIGURE 1:**
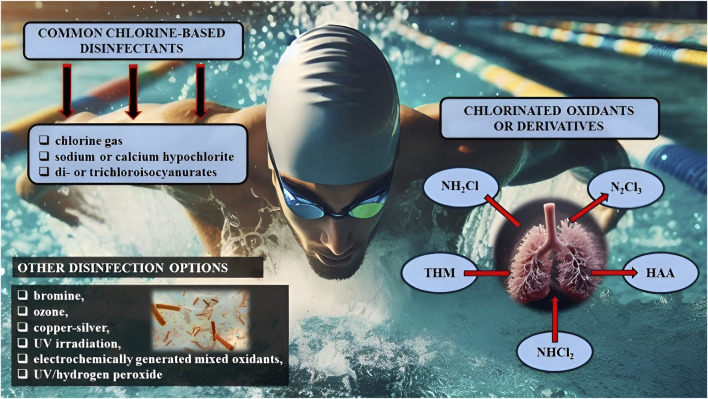
Chlorine-based disinfectants commonly used in indoor pools and swimmers’ exposure to chlorinated oxidants/derivatives that can be inhaled, ingested or absorbed through the skin. Note: NH_2_Cl, monochloramines; NHCl_2_, dichloramines; N_2_Cl_3,_ trichloramines; THM, trihalogenomethanes; HAA, haloacetic acid; UV, ultraviolet.

The World Health Organization (WHO) recommends adequate water disinfection to prevent microbial proliferation in swimming pools. Chlorine-based disinfectants (chlorine gas, sodium or calcium hypochlorite, di- or trichloroisocyanurates) are commonly used in indoor pools due to their effectiveness and lower relative cost, despite possibly leading to unwanted effects (Couto et al., 2021). Although there are several disinfection options available, such as bromine, ozone, copper-silver, UV irradiation, electrochemically generated mixed oxidants, and UV/hydrogen peroxide (Figure 1), chlorination still remains the most widely used method to disinfect pool water (Westerlund et al., 2019; Couto et al., 2021).

When reacting with organic or inorganic matter (such as urea, sweat, dandruff, and skin flakes) chlorine produces chlorinated oxidants or derivatives (CBPs with high redox potentials) which can be inhaled, ingested or absorbed via the skin (which may represent a source of muco-cutaneous symptoms). These include monochloramines (NH_2_Cl), dichloramines (NHCl_2_) and trichloramines (N_2_Cl_3_), as well as organohalogen compounds such as trihalogenomethanes (THM) and haloacetic acid (HAA) (see Figure 1; Cavaleiro et al., 2018; Jacobs et al., 2007; Kanikowska et al., 2018).

Scientific research has demonstrated that swimming pool water can possess mutagenic properties, with varying degrees of potency reported in different studies. Many disinfection by-products (DBPs) have carcinogenic and genotoxic properties, and exposure to DBPs have been linked to reproductive and neurotoxic adverse effects in animal studies. Human epidemiological studies have shown that exposure to DBPs can increase the likelihood of respiratory adverse effects and bladder cancer, although the association between DBPs and other health effects is still uncertain (Manasfi et al., 2017). Epidemiological data about the risk of cancer are still controversial. However, numerous publications highlight a toxic risk, especially the risk of allergy and respiratory symptoms, for babies and elite swimmers (Florentin et al., 2011).

Trichloramine (NCl_3_), an inorganic chloramine (0.02 mg/L in water as Cl_2_), is considered one of the most toxic substances–an easily volatile substance with a characteristic odour that occurs as DBP in the air of chlorinated indoor swimming pools from reactions of nitrogenous compounds with chlorine (Schmalz et al., 2011). Trichloramine in higher concentrations causes acute irritation of the respiratory tract mucosa (Bernard et al., 2005). Seys et al. (2015a) described a situation in which, as a result of an accidental increase in the concentration of trichloramine in the air, people in indoor swimming pools complained of coughing, shortness of breath, watery eyes and nasal congestion. Twenty-two of the 26 subjects were found to have bronchial hyperreactivity during a histamine provocation test (Kanikowska et al., 2018).

The level of CBPs above the water surface is influenced by factors such as chlorine dosage, air temperature, pool area ventilation, and swimmer/bather load. Swimmers are exposed to these by-products when inhaling air and aerosols right above the water surface, where is their highest concentration (Schmalz et al., 2011). In regular swimmers, at a ventilation rate of 22 L/min, there is a switch from nasal to mouth breathing. Consequently, the air inhaled undergoes less filtration by the nasal cavity and CBPs are able to travel further into the lungs (Lomax, 2016; Kanikowska et al., 2018). A study by Voisin et al. (2014) involving 196 preschool-aged children found that regular swimming in a pool with chlorinated water before the age of three was associated with higher serum allergen-specific IgE (sIgE) antibody concentrations for house dust mites and higher nitric oxide concentrations in exhaled air between the ages of five and seven, a risk factor for the development of bronchial asthma and rhinitis. A large Swedish population-based study involving 1,652 children aged 11–12 found that children who visited the pool more than once a week were more likely to develop bronchial asthma than those who swam less often. The risk was higher only in children who were allergic to aeroallergens, as confirmed by prick skin tests (Andersson et al., 2015). Even in adults who swam recreationally, a higher incidence of new-onset asthma was observed in a study involving 1136 adults, and the development of the disease was related to the cumulative number of hours spent in indoor pools over a lifetime (Ferrari et al., 2011).

In a study by Carbonelle et al. (2002) transient bronchial epithelial damage was observed after an hour-long stay in an environment containing chlorine compounds in children and adults who swam recreationally, as well as in those who passively inhaled air containing chlorine compounds. Such recurrent damage is most likely related to oxidative stress and causes inflammation, which then leads to the development of sensitization to aeroallergens. In most competitive swimmers, cessation of professional swimming training results in a decrease in bronchial hyperresponsiveness and airway inflammation. However, it is still not known whether this is fully reversible phenomenon, and over what period of time the decrease occurs (Bougault et al., 2011). Sun et al. (2022) presented a study that included 2359 adolescents aged 12–19 years with measured blood concentrations of chloroform (trichloromethane, TCM; bromodichloromethane, BDCM; dibromochloromethane, DBCM; and bromoform: tribromomethane, TBM) from the National Health and Nutrition Examination Survey 2005–2012. The results have shown that blood DBCM concentrations were associated with a higher risk of ever asthma among all adolescents. They also found positive relationships between blood brominated THM concentrations and risk of ever asthma and between blood DBCM and brominated THM concentrations and risk of current asthma among adolescents with tobacco smoke exposure. However, in a study performed by Valeriani et al. (2017), different conclusions have been made. Their systematic review and meta-analysis led to the assumption that swimming in childhood does not increase the likelihood of doctor-diagnosed asthma. Based on this meta-analysis review, the association of the disease with indoor pool attendance is still unclear. There are other potentially hazardous compounds from pharmaceuticals and personal care products (PPCPs) that are present in swimming pools (Celeiro et al., 2018). A few toxicological studies suggest that some organic UV filters have significant estrogenic and antithyroid effects (Kinnberg et al., 2015; Schmutzler et al., 2007) and they can bioaccumulate. Unfortunately, CBDs may react with the organic UV filters and may contribute to the formation of chlorinated DBPs (Santos et al., 2012). Therefore, considering the potential harmful effects of UV filters in swimming pool waters, the development of effective processes that lead to a complete removal of UV filters is necessary.

Not only the pool water contains harmful substances causing pathological mechanisms, but also the air in the swimming pool is saturated with nitrogen trichloride, which causes so-called occupational asthma in water rescuers and swimming instructors who spend long hours at the pool inhaling multiple substances (Cavaleiro et al., 2018). Thickett et al. (2002) presented three well-documented cases of occupational asthma in swimming-pool workers. Two of these individuals had a positive bronchial provocation test to chloramine in the laboratory, there is little doubt that these people had occupational asthma. However, the exact cause of the asthma in these individuals is still unknown. While it may be due to immunological sensitization, the fact that they reacted to low levels of trichloramine suggests that it may not be a purely irritant effect. A pure irritant effect was, therefore, considered less likely as the subjects had no increased bronchial reactivity to histamine. Irritant-induced asthma caused by chronic low-level exposure to irritant agents is a controversial topic with limited epidemiological and experimental support.

There are no population-based studies comparing the impact of using swimming pools with alternative water disinfection agents: ozone, UV radiation, bromine and salt, as well as silver and copper ions. The optimal microbiological and chemical method is ozonation of water, but due to its higher cost, this method is rarely used, nor is it completely free of toxic by-products. When using pools with chlorinated water, air circulation is crucial, as it disperses toxic compounds suspended in the air. It is also important to use the smallest effective amount of chlorine disinfectant, for pool users to wash their bodies thoroughly before swimming, and to use swimming caps to limit the delivery of organic compounds into the pool. Asthma treatment in competitive swimmers should be based on prevention of exposure to chlorine substances. The level of nitrogen trichloride should be constantly monitored, fresh air supply should be ensured and the proper air and water temperature should be maintained (Belda et al., 2008; Cavaleiro et al., 2018; Kanikowska et al., 2018). Some recent advances in chlorination and DBPs, their types, DBPs guidelines, and methods for removing DBPs have been described (Gani et al., 2024). Two basic approaches were identified to mitigate the risks associated with the effects of DBPs on the human body: [1] reducing DBP formation by the use of chlorine dioxide (ClO_2_), which has a higher oxidation potential than chlorine and can therefore more effectively oxidize organic matter in water; [2] the use of monochloramine, a less reactive and less volatile form of chlorine, that can be more stable in water and reduce DBP formation. These and other advances in chlorine applications can contribute to safer and more effective water treatment.

### Effects of swimming on spirometric parameters and bronchial hyperresponsiveness

Spirometry is the main respiratory function assessment test, as a standardized and reproducible method for estimating the respiratory mechanism and pulmonary ventilation (Lazovic-Popovic et al., 2016). There have been several studies conducted to examine the effects of swimming on spirometry parameters. For instance, a study conducted by Wicher et al. (2010) focused on children and adolescents with mild persistent asthma. The participants were divided into two groups: a swimming group and a control group. Both groups received standard asthma medication, while the swimming group also underwent a swim training program consisting of 2 weeks^−1^ classes over a 3-month period. The results showed that the participants in the swimming group experienced a significant improvement in bronchial hyperresponsiveness compared to the control group. This was measured by an increase in the provocative concentration of methacholine causing a 20% fall in forced expiratory volume in 1 s (FEV1) values. Additionally, the swimming group showed improvement in the elastic recoil of the chest wall.

Another interesting study conducted systematic review and meta-analysis to examine the effects of physical activity on lung function and quality of life (QoL) in asthmatic children (Jing et al., 2023). Randomised controlled trials were included and two reviewers independently conducted the inclusion screening, data extraction and bias assessment. A total of 9 studies were included in this review after 3,919 articles were screened. The authors found that physical activity significantly improved forced vital capacity (FVC) and forced expiratory flow between 25% and 75% of forced vital capacity (FEF25-75), which are measures of lung function. However, there was no significant improvement in FEV1 or fractional exhaled nitric oxide (FeNO), which indicates airway inflammation. Physical activity also had a positive impact on the QoL of asthmatic children.

Various studies using spirometry tests revealed that competitive swimmers had a mean baseline FEV1 and FVC of approx. 110% of predicted values. Airway obstruction was observed in 12% of the swimmers included in the study. Interestingly, with the exception of two individuals, all swimmers exhibiting airway obstruction were males. In addition, swimmers who swam a distance of more than 2,000 m reported significantly more respiratory symptoms compared to the other subjects (Päivinen et al., 2010; Päivinen et al., 2021).

In the study of Fiks et al. (2012) 1,116 young swimmers between 8 and 17 years of age completed the questionnaire and underwent spirometry at rest. Among all of the participants, 254 individuals (22.7%) were identified to have airflow obstruction (FEV1/FVC <80%).

### Exercise-induced effects on pulmonary function (swimming *versus* other types of physical activity)

In general, competitive swimmers achieve larger lung volumes and higher functional cardiorespiratory system capacity compared to other athletes (Doherty and Dimitriou, 1997). However, no large-scale studies have been conducted to analyse in detail the effects of specific sports on pulmonary function, including asthma. Recently, the prospective study conducted by Malewska-Kaczmarek et al. (2022) examined the impact of different types of physical activity, including swimming. The research involved a group of Polish Caucasian children who were involved in sports activities at schools and clubs. The study considered a range of sports starting with football, horse riding, tennis, dance, athletics, cycling, martial arts, gymnastics, floorball, basketball, volleyball, handball, and swimming. Children with a diagnosis of chronic respiratory system diseases other than asthma were excluded, as were those who did not cooperate during the lung tests or had contraindications for the planned tests. Additionally, children with asthma who were undergoing treatment were asked to refrain from using long-acting beta-2 mimetics (LABA) for 24 h before their scheduled visit.

Spirometry was performed to measure lung function, a pulmonary resistance test, a measurement of fractional exhaled nitric oxide (FeNO) concentration to assess airway inflammation, and a standardised exercise test to evaluate response to physical exertion. They also performed a treadmill exercise challenge, and the parameter analysed was the percentage of FEV1, which represents the difference between the highest and lowest FEV1 achieved before and after the exercise challenge. The findings showed that swimmers had the highest FEV1 values, followed by indoor athletes. This suggests that swimming and certain types of physical activities can positively impact lung capacity, with swimmers exhibiting the highest values compared to other athletes (Lazovic-Popivic et al., 2016). Overall, these studies indicate that swimming and physical activity in general can have beneficial effects on spirometry parameters, such as bronchial hyperresponsiveness, lung function (FVC and FEF25-75), and QoL in asthmatic children. However, more research is needed to determine the specific effects of swimming compared to other types of physical activity on lung capacity and asthma control. The data on the differences in the risk of respiratory dysfunction according to the level of sports training are also of interest. Competitive swimmers who had the highest exposure to swimming pool environments, reported significantly more respiratory symptoms compared to fitness and occasional swimmers. Overall, 18% of the swimmers studied reported respiratory symptoms, with competitive swimmers being the most affected group. Moreover, competitive swimmers reported three times as many respiratory symptoms as other activity groups in swimming halls (Päivinen, et al., 2021).

**FIGURE 2:**
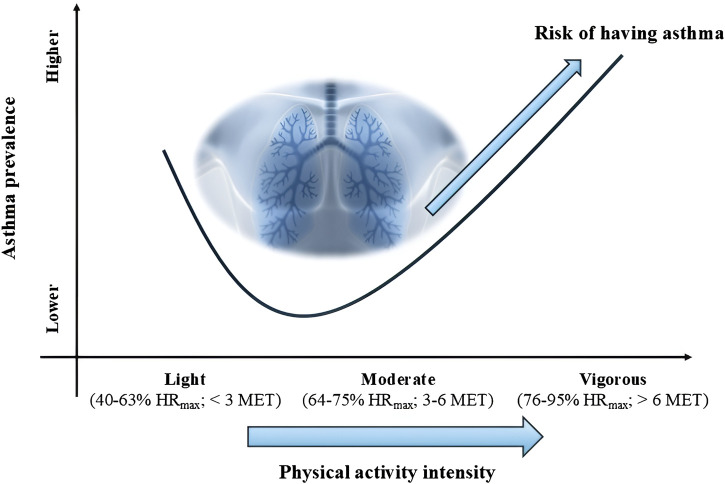
Dose-response relationship between physical activity intensity and asthma risk. Note: HR_max_, heart rate maximum; MET, metabolic equivalent of task.

It is currently accepted that strenuous (76%–95% HR_max_; >6 MET) exercise increases the risk of developing asthma, assuming a dose-response relationship between physical activity and EIA/EIB risk (see Figure 2). This relationship is curvilinear (“U”-shaped curve), showing that moderate-intensity exercise (64%–75% HR_max_; three to six MET) results in a lower risk of asthma compared to low-intensity exercise (40%–63% HR_max_; <3 MET) and high-intensity exercise (especially endurance and interval training) (Del Giacco et al., 2015).

According to the study of Carlsen et al. (2008) asthma and allergies are becoming increasingly prevalent issues among actively competing athletes, particularly in elite endurance-trained athletes, where EIA or EIB is frequently reported. The prevalence of asthma and bronchial hyperresponsiveness (BHR) is significantly higher in endurance-trained athletes in sports such as swimming, cycling and triathlon. According to Fitch et al. (2012), over 17% of Olympic swimmers and 13% of synchronised swimmers have asthma and/or BHR. Additionally, other forms of pollution can also impact athletic performance. Therefore, epidemiological studies in athletes provide substantial indirect evidence suggesting that various factors such as pollen exposure in summer athletes, cold air and respiratory infections in skiers, chlorine exposure in swimmers, and fine particles in ice rinks can adversely affect respiratory health in competitive athletes.

Heavy endurance swimming training, similarly to high-intensity interval training, especially under adverse conditions, stresses the airway mucosa, leading to inflammatory changes (i.e., higher levels of eosinophils and FeNO) as observed in induced sputum in competitive swimmers (Helenius et al., 1998). Consequently, the combination of intense and repeated physical endurance training over extended periods, along with suboptimal environmental conditions, may contribute to the development of asthma and BHR in elite athletes. Larsson’s et al. (1998) study demonstrated that cold air inhalation increased the number of inflammatory cells in bronchoalveolar lavage. In children, Bernard et al. (2003) found a correlation between the time spent in swimming pools during early childhood and the development of asthma, as well as signs of lung involvement indicated by increased serum levels of surfactant proteins.

### Bronchodilators

Bronchial asthma is a chronic inflammatory condition of the lower airways characterised by breathlessness, bronchial obstruction and coughing. Various combinations of the involved factors determine and modify the final clinical phenotype/endotype of asthma (Jesenak et al., 2017). Exercise-induced symptoms are commonly referred to as transient bronchoconstriction (Jayasinghe et al., 2015). The best way to verify the influence of the bronchodilators on the respiratory system is to examine the swimmers who systematically attended the swimming pool, especially professional long-distance swimmers. Inhaled short-acting β2-agonists (SABAs) are frequently needed and strongly suggested as pre-treatment before competition. If insufficient, long-acting β2-agonists (LABAs) and leukotriene antagonists may be added (Carlsen et al., 2008). Based on current anti-doping regulations, competitive swimmers are permitted to use specific inhaled β2-adrenergic agonists, both short- and long-acting, such as formoterol, salbutamol, salmeterol, terbutaline, and inhaled corticosteroids (ICS), in dosages precisely determined in the Therapeutic Use Exemptions (TUEs). In cases of properly treated and controlled asthma, these substances, at the dosages defined by World Anti-Doping Agency (WADA), effectively suppress the symptoms of the disease without posing a risk to the swimmer’s health (Courteix et al., 1997; Bougault et al., 2009; Mountjoy et al., 2015). The vast majority of studies show that the use of the aforementioned substances by healthy non-asthmatic athletes does not increase physical performance (e.g., endurance and speed) (Zwick et al., 1990; Momas et al., 1993; Murray, 2010), with the exception of oral short-term administration of salbutamol, which enhances voluntary muscle strength in man (Martineau et al., 1992). On the contrary, recent systematic review and meta-analysis of randomized controlled trials showed that non-asthmatic subjects can improve sprint and strength performance by using β2-adrenergic agonists (Riiser et al., 2020). However, it should be noted that the improvement was observed for maximal physical performance lasting 1 min or shorter.

From a physiological standpoint, there is no justification for including β2-adrenergic agonists on the WADA Prohibited List, as there is no strong evidence to suggest ergogenic (performance-enhancing) effects in competitive athletes, such as improved endurance and speed, unless they have asthma. To summarize, the most up-to-date systematic reviews and meta-analyses demonstrated that β2-adrenergic agonists do not affect sports performance in healthy athletes (including swimmers) regardless of type, dose, administration route, duration of treatment or the performance level of the participants.

## Summary and outlook

This narrative review takes an approach to examine the complex relationship between swimming and pulmonary function in chlorinated pools, with a particular focus on the impact of asthma. The scientific literature confirms that chlorine exposure to areas where swimming exacerbates asthma symptoms and increases airway hyperesponsiveness (AHR). A series of studies confirms the increased respiratory symptoms in swimmers, suggesting adverse effects of chlorine compounds such as trichloramine (Zwick et al., 1990; Helenius et al., 2002; Gomà et al., 2017; Nitter and Svendsen, 2020). This finding is important, highlighting the need to reevaluate pool disinfection strategies and enhance safety measures for swimmers’ respiratory health. Notably, the confirmed association between chlorinated pool exposure and elevated serum sIgE levels, as reported by Voisin et al. (2014), suggests potential association between allergens, chlorine exposure and the development of asthma. Additionally, available evidence suggests that factors like the frequency of pool visits and the time spent in indoor pools are important in understanding the respiratory health of swimmers. This perspective, aligning with the findings of Ferrari et al. (2011), enhances our knowledge about the environmental factors that can influence pulmonary dysfunction (e.g., bronchial asthma) in swimmers. Moreover, the review is consistent with studies by Bernard et al. (2006) and Font-Ribera et al. (2009), which reported increased respiratory problems in swimmers exposed to chlorinated environment. This comparison places these issues in the larger context of current research, highlighting the unique challenges swimmers face.

Swimming provokes multiple psychophysiological effects, including blood, hormonal, enzymatic, cardiovascular and energetic adaptations (Costa et al., 2015). However, this review also highlights the respiratory risks associated with swimming, particularly due to chlorine derivatives. Chronic exposure to these compounds can disrupt the normal functioning of the respiratory system, leading to increased muscle constriction in the airways and worsening asthma symptoms by promoting inflammation. While occasional or low-level exposure to chlorine might not be harmful, regular swimmers, especially those at competitive levels, are at a higher risk of developing respiratory disorders. Because these potential risks of exposure to CBDs must be balanced against the benefits of swimming and the risk of microbial infections in pools, we recommend better pool management and regular health checks for swimmers. Chlorine by-products are primarily responsible for conditions like swimming pool-induced rhinitis, asthma and AHR (Bougault et al., 2009). Interestingly, the reduction of these symptoms in swimmers who reduce training volume and intensity suggests that the negative effects on airway function may be reversible (Helenius et al., 2002). For these reasons, it is crucial to develop effective respiratory protection strategies, including medical interventions and modifications to the pool environment. In athletes, asthma treatment aims to enable high-intensity performance without asthmatic symptoms while strictly adhering to WADA regulations. Athletes with asthma may use inhaled β2-blockers and corticosteroids in accordance with TUE specifications. Practical steps such as reducing chlorine use, ensuring proper hygiene before swimming and using swim caps can minimize the risks. Research should also explore safer alternatives to CBDs, such as ozonation, and improved ventilation to reduce air pollutants.

Further research should aim to understand the combined effects of exercise and inhalation of chlorine by-products and the direct effect of pool chemicals (chloramines) as potential inducers of epithelial damage and airway inflammation.
